# Current knowledge about early childhood caries in the gulf cooperation council with worldwide reflection: Scoping review of the scientific literature (2010–2021)

**DOI:** 10.1371/journal.pgph.0001228

**Published:** 2023-01-12

**Authors:** Asmaa Othman Alkhtib, Hasaan G. Mohamed

**Affiliations:** 1 Managing Director Office, Primary Health Care Corporation, Doha, Qatar; 2 Dentistry Department, Primary Health Care Corporation, Doha, Qatar; Egas Moniz Cooperativa de Ensino Superior CRL, PORTUGAL

## Abstract

Early childhood caries (ECC) is one of the most prevalent chronic childhood diseases affecting the primary teeth of children younger than 6 years of age. The disease etiology is complex and includes social, biological, and dietary factors. This review aims to explore the knowledge of ECC prevalence globally and locally within the Gulf Cooperation Council (GCC) countries during the years 2010–2021. Another aim is to explore oral health promotion programs with more focus on the GCC region. A search was conducted in PubMed, Medline, Scopus, the Cochrane Collaboration database, and Google Scholar to identify relevant studies published between 2010 and 2021 using specific keywords. Studies that utilized both the World Health Organization criteria and International Caries Detection and Assessment System for dental caries assessment were included. The included studies indicated considerable variation in the reported prevalence of ECC. While developed countries show low prevalence, countries in the GCC and other Arab countries show a high prevalence of ECC. Many oral health promotion programs were identified globally including oral health education, nutritional programs, the use of fluoride and pit-and-fissure sealants, and inter-professional population-based oral health promotion and prevention programs such as school-based oral health programs, motivational interviewing, and anticipatory guidance. ECC remains a significant problem in many parts of the world including the GCC region. Oral health prevention programs have been established within the GCC region. Nevertheless, the GCC region has some unique characteristics that need to be investigated to contextualize the western model of the dynamics of ECC prevention and promotion programs locally.

## Introduction

Dental caries is a major public health concern for children worldwide and exerts a huge socioeconomic burden on families and society, especially when affecting young children [1]. Early Childhood Caries” ECC is defined by the American Academy of Pediatric Dentistry as “*The presence of one or more decayed (cavitated or non-cavitated)*, *missing due to caries or filled tooth surfaces in any primary tooth in a child under the age of six*” [2]. Several terminologies are used to describe the condition such as nursing bottle caries, nursing caries, rampant caries, baby bottle caries, baby bottle tooth decay, milk bottle syndrome, and prolonged nursing habit caries [3].

ECC is one of the most prevalent chronic childhood diseases affecting large numbers of children globally [4]. It is a multifactorial disease with specific characteristics and clinical presentation as it often affects tooth surfaces that are less prone to caries development. The disease results from the interaction of factors that include cariogenic microorganisms, exposure to fermentable carbohydrates through inappropriate feeding practices, and a range of social variables. The clinical presentation can extend from localized pain to include infections, abscesses, difficulty in chewing, malnutrition, delays in growth and development, gastrointestinal disorders, difficulty in sleeping, and sometimes hospitalizations and emergency room visits [3]. Another impact to be considered is the loss of school hours, diminished quality of life, and the negative influence on caregivers [5].

Although ECC is preventable and reversible within the early stages of the disease, management of ECC may require extensive restorative treatment, premature dental extraction, and subsequent need for space maintenance and orthodontic treatment in adult life. Moreover, children at this young age may not be cooperative enough to allow dentists to perform such intense treatment plans, which necessitates the utilization of sedation or general anesthesia [3].

Several studies indicated that ECC mostly affects malnourished children and those living in underprivileged communities with low socioeconomic status. Nevertheless, high ECC prevalence has been observed in communities with high socioeconomic status including the Gulf region [6, 7]. The Gulf Cooperation Council (GCC) comprises six countries: Bahrain, Kuwait, Oman, Qatar, Saudi Arabia, and the United Arab Emirates. Although the GCC countries are considered high-income countries where governments provide the majority share of the health budget, early childhood caries remains a significant problem [8–11]. In Qatar, the ECC prevalence was 89% [8], and in The United Arab Emirates (UAE) and The Kingdom of Saudi Arabia (KSA), the ECC prevalence was 83% [12] and 73% [9] respectively.

It is clear that the western model of the dynamics of early childhood caries and the effect of socioeconomic status cannot be applied directly to the GCC region [13]. This region has some unique characteristics that need to be researched and unpacked in order to understand the real influence of the social structure on the oral health of young children.

This review aims to: a. explore and summarize the knowledge about ECC prevalence and its burden globally with more focus on the GCC countries during the past decade, b. identify the available oral health promotion interventions to prevent ECC in different geographical settings, and c. provide useful insight for decision-makers on effective and efficient ECC prevention programs which can be adapted within their local settings.

## Materials and methods

This literature review is a scoping review, conducted by two researchers with expertise in the field. The methodology of conducting this review was adapted from Peters MDJ (2020) [14] (S1 Checklist).

### Review questions

This review was guided by the following questions: “*what is the reported ECC prevalence and mean decayed*, *missing*, *and filled teeth index (dmft) in different geographical areas around the world in comparison to the GCC region*?”, and *“What strategies have been implemented around the world to prevent ECC and to what extent they were successful*?”. These questions shall provide insight into the current situation of the GCC countries compared to the rest of the world and inform policymakers on the future steps to be taken to prevent the disease.

### Inclusion criteria

The literature search focused on studies reporting on ECC prevalence and prevention programs with the following specifications:

#### Participants

This review included studies that reported on dental caries prevalence in children between the age of 0 and 6 (as per the ECC definition). Studies involving medically compromised children, children with other health issues, and children as minority groups (e.g., refugees) were excluded.

#### Concept

Studies that utilized both the WHO criteria [15] and International Caries Detection and Assessment System (ICDAS) [16] for dental caries assessment were included. The dmft index was captured within the identified ECC prevalence studies if reported. The review also included studies on ECC prevention with a clear description of the target population, preventative interventions, and outcomes.

#### Context

The literature search focused on primary studies from all countries reporting on ECC prevalence in the last decade (between 2010–2021). Hence the results were reported based on the geographical location of the included studies. Moreover, an exploratory search has been conducted utilizing relevant keywords highlighted in Table 1 to identify studies on ECC prevention programs. These studies were not included in the main results section. Nevertheless, the findings of those studies were included in the discussion section.

**Table 1:** Inclusion and exclusion criteria and keywords for the literature search.

Inclusion criteria	Exclusion criteria	Keywords
• Studies that investigated oral health promotion research in general • Studies that evaluated oral health promotion interventions for healthy preschool children • Studies reporting caries prevalence and/or caries experience of primary teeth of children aged 0 to 6 years • Governmental policies and documents regarding the oral health of preschool children • WHO documents regarding oral health policies, conferences, and workshops pertaining to preschool children. • Studies published in English from 2010–2021	• Studies involving medically compromised children or children with other health issues • Studies published in other languages besides English • Studies involving Children as a minority • Duplicated reports or studies using the same data	• Caries, early childhood caries, nursing caries • Preschool child (ren), infant, oral health, dental, teeth, caries, decay, risk factors, social determinants, quality of life, intervention, management, prevention fluoride, mother, caregiver, primary health care, world health organization, GCC countries, Qatar • Nursing bottle caries, rampant caries, baby bottle caries, baby bottle tooth decay, milk bottle syndrome, and prolonged nursing habit caries.

#### Types of evidence sources and search strategy

The literature search was conducted on the major health research databases: PubMed, Medline, Scopus, and the Cochrane Collaboration. An additional search in the Google Scholar database was conducted to enable a broader view of the available government policies. Moreover, the World Health Organisation (WHO) documents were checked regarding oral health policies, conferences, and workshops of relevance. In addition, the bibliographies of the located articles were checked for additional relevant references. Exclusion criteria included studies published in languages other than English, case studies, and case reports, studies included children older than 6 years of age, review articles, and meta-analyses. The inclusion and exclusion criteria and keywords are detailed in Table 1. Articles were reviewed independently by two researchers.

## Results

**Fig 1:**
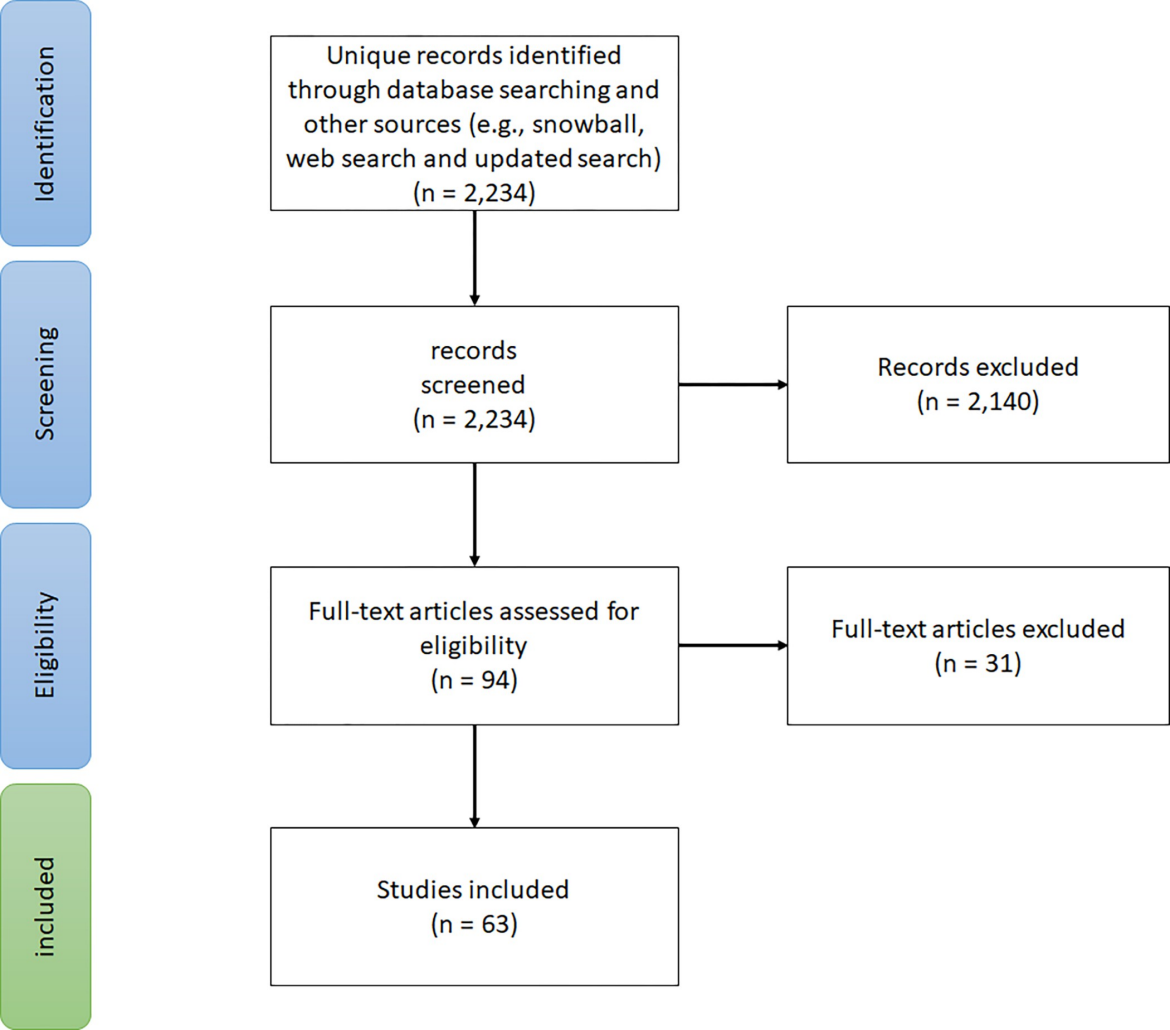
Flowchart of the study selection process (adapted from Peters, Micah DJ et al, (2015) [17].

The initial search of the databases and other sources was conducted in August 2021 and resulted in 2,234 studies. the initials screening excluded 2,140 studies after reviewing the titles and abstracts and 94 studies were included in the full-text review for eligibility. Of the 94 articles, 63 studies were included in this review. The process of records identification and selection are summarized in Fig 1.

The extracted data from the included articles were charted in table format and categorized based on the geographical location to serve the study objectives. The table includes the authors, year of publication, the country where the study was conducted, the age range and number of the study participants, the ECC prevalence (as percentage), and the mean dmft index (if reported) (Table 2).

**Table 2:** Included studies reporting on dental caries prevalence and experience in primary teeth of children 0–6 years.

Authors/year	Country	Age	Sample size	Prevalence (%)	dmft
**GCC & other Arab countries**					
Alkhtib A, et al, (2016) [8]	Qatar	4–5 y	250	89.2%	3.3
Alotaibi F, et al, (2017) [9]	KSA	3–6 y	224	72.7%	3.69
Al-Meedani LA, et al, (2016) [18]	KSA	3–5 y	388	69%	3.4
El Nadeef MA, et al, (2010) [12]	UAE	5 y	1,340	83%	5.1
Kowash MB, et al, (2017) [10]	UAE	4–6 y	540	74.1%	3.07
Shalan HM, et al, (2018) [19]	Egypt	3–6 y	1,000	61.4%	2.93 (males), 2.33 (females)
Aziz Z, (2014) [20]	Palestine	4–5 y	1,376	76%	2.46
Chedid NR., et al, (2011) [21]	Lebanon	2–4 y	99	74.7%	-----
Elelmi Y, et al, (2021) [22]	Tunisia	3–5 y	393	49.9%	1.42
Elidrissi SM, et al, (2016) [23]	Sudan	3–5 y	553	52.4%	2.3
Qadri G, et al, (2012) [24]	Syria	3–5 y	400	70%	4.25
**Europe**					
Boustedt K, et al, (2020) [25]	Sweden	5 y	336	18.9%	----
Strömberg U, et al, (2012) [26]	Sweden	3–6 y	10,927	11.4%	----
Nobile CG, et al, (2014) [27]	Italy	3–6 y	515	19%	2.68
Congiu G, et al (2014) [28]	Italy	1.5–5 y	544	15.9%	----
Colombo S, et al, (2019) [29]	Italy	0–71 months	3,000	8.2%	----
Ozer S, et al, (2011) [30]	Turkey	3–6 y	226	46.9%	2.87
Doğan D, et al, (2013) [31]	Turkey	8–60 months	3,171	17.3%	----
Igic M, et al, (2018) [32]	Serbia	3–6 y	250	52.8%	2.38
Oulis CJ, et al, (2011) [33]	Greece	5 y	1,209	36%	1.77
Šačić L, et al, (2016) [34]	Federation of Bosnia and Herzegovina	3–5 y	165	83%	6.79
Begzati A, et al, (2010) [35]	Kosovo	1–6 y	1,008	17.36%	5.8
Baggio S, et al, (2015) [36]	Switzerland	36–71 months	856	24.8%	----
Slabšinskienė E, et al, (2010) [37]	Lithuania	3 y	950	50.6%	2.1
Olczak-Kowalczyk D, et al, (2020) [38]	Poland	3 y	656	53.8%	2.4
**Asia**					
Prakash P, et al, (2012) [39]	India	8 and 48 months	1,500	27.5%	0.85
Singh S, et al, (2012) [40]	India	3–5 y	717	40%	1.89
Chugh VK, et al, (2018) [41]	India	3–6 y	425	47.3%	----
Srikanth Koya S, et al, (2016) [42]	India	2–6 y	1,897	41.9%	1.51
Henry JA, et al, (2017) [43]	India	0–3 y	1,486	40.6%	----
Gopal S, et al, (2016) [44]	India	3–6 y	447	27.3%	2.36
Stephen A, et al, (2017) [45]	India	3–6 y	2,771	16%	----
Gaidhane AM, et al, (2013) [46]	India	3–5 y	330	31.8%	----
Amanlou M, et al, (2011) [47]	Iran	3–6 y	205	49.3%	0.99
Shaghaghian S, et al, (2018) [48]	Iran	3–6 y	453	69.9%	3.88
Toutouni H, et al, (2015) [49]	Iran	2–3 y	239	61.1%	----
Nishino M, et al, (2020) [50]	Mongolia	0–5	598	82.6%	----
Senesombath S, et al, (2010) [51]	Laos	36–47 Month	400	82%	5.5
Zhou Y, et al, (2011) [52]	China	2 y	394	27.7%	3.62
Zhang, et al, (2014) [53]	China	5 y	723	85%	5.8
Wang Z, et al, (2019) [54]	China	6 y	4,936	87.7%	6.04
Li Y, et al, (2017) [55]	China	3–5 y	1,727	78.2%	5.61
Jiang YY, et al, (2017) [56]	China	2–5 y	2,829	53.3%	2.12
Kato H, et al, (2017) [57]	Japan	3 y	6,315	14.7%	----
Duangthip D, et al, (2019) [58]	Hong Kong	3–5 y	1,204	46%	2.1
Kumarihamy SL, et al, (2011) [59]	Sri Lanka	1–2 y	422	32.2%	2.01
Perera PJ, et al, (2012) [60]	Sri Lanka	2–5 y	410	68.8%	----
Chanpum P, et al, (2020) [61]	Thailand	9–18 months	513	42.5%	1.07
Peltzer K, et al, (2015) [62]	Thailand	3 y	597	68.5%	----
Nirunsittirat A, et al, (2016) [63]	Thailand	3–4 y	556	88.1%	14.2
Khanh LN, et al, (2015) [64]	Vietnam	1–6 y	593	74.4%	5.87
Turton B, et al, (2019) [65]	Cambodia	0–4 y	3,985	56.6%	3.5
Kubota Y, et al, (2020) [66]	Cambodia	1.5–3 y	121	54.5%	2.81
**Central /South America**					
Dabiri D, et al, (2016) [67]	El Salvador	0–6 y	886	58%	----
Piva F, et al, (2017) [68]	Brazil	3–4 y	119	89.9%	-----
Percival T, et al, (2019) [69]	Trinidad and Tobago	3–5 y	342	50.3%	2.83
Castillo JL, et al, (2019) [70]	Peru	3–5 y	2,195	76.2%	5
**Africa**					
Musinguzi N, et al, (2019) [71]	Uganda	3–5 y	432	48.6%	2.04
Rwakatema DS, et al, (2010) [72]	Tanzania	3–5 y	372	30.1%	0.95
Njoroge NW, et al, (2010) [73]	Kenya	3–5 y	336	59.5%	2.46
Iyun OI., et al, (2014) [74]	Nigeria	3–5 y	540	23.5%	0.65
Folayan MO, et al, (2015) [75]	Nigeria	6–71 months	497	6.6%	0.15
**Australia**					
Devenish G, et al, (2020) [76]	Australia	2–3 y	1,039	10.6%	----

### Trends of ECC prevalence

It can be seen from Table 2 that studies on the prevalence of ECC globally and regionally report great variation. While developed countries show low prevalence, countries in the GCC and other Arab countries show a high prevalence of ECC. Several studies from the GCC region documented a high prevalence of ECC in children. The highest prevalence was reported in Qatar (89% in 4–5 year-olds, followed by The United Arab Emirates (UAE) (83% in 5 year-olds & 74% in 4–6 year-olds), while the prevalence in the Kingdom of Saudi Arabia (KSA) was 69% in 3–5 year-olds and 73% in 3–6 year-olds. Reports from other Arabic countries showed similar trends of high prevalence with the highest being reported from Palestine (76% in 4–5 year-olds) followed by Lebanon (75% in 2–4 year-olds), Egypt (61% in 3–6 year-olds), Sudan (52% in 3–5 year-olds) and the least prevalence among the Arabic countries was reported in Tunisia (50% in 3–5 year-olds). There was a considerable variation in the reported prevalence of ECC from studies conducted in Europe. The highest prevalence was observed in a study from The Federation of Bosnia and Herzegovina (83% in 3–5 year-olds) while the lowest prevalence of 8% in 0–71 month-olds was reported in Italy in 2019. A big variation in the reported prevalence of ECC was also observed in studies from Asia. China reported the highest prevalence of 88% in 6 year-olds and 85% in 5 year-olds and the lowest prevalence in Asia was reported in Japan with a prevalence of 15% in 3 year-olds.

In Central & South America, the highest prevalence of ECC was observed in Brazil (90% in 3–4 year-olds) while the lowest prevalence was reported in Trinidad & Tobago (50% in 3–5 year-olds). The highest prevalence of ECC in Africa was reported in Kenya (60% in 3–5 year-olds), while Nigeria showed a relatively low prevalence of ECC compared to other studies from the same region; 24% in 3–5 year-olds and 7% in 6–71 month-olds. Only one recent study from Australia was included in this review with a reported prevalence of 11% in 2-3-year-old.

### ECC oral health prevention programs

Table 3 below indicates the different ECC oral health preventative programs highlighted in this review and their reported outcomes

**Table 3:** ECC oral health prevention programs and their reported outcomes.

Authors/year	Country	Target population	Sample size	Preventative measure	Outcome
Alsumait, A., et al. (2019) [77]	Kuwait	Primary school children, 11–12 y	440	School school-based oral health program including twice-a-year applications of fluoride varnish and fissure sealants if needed. Mothers had, at least, one oral health education session	Positive impact on children’s caries level with no significant impact on mothers’ knowledge, attitude, practice, or OHRQoL.
AlKlayb, S. A., et al. (2017) [78]	KSA	Mothers of children below 6 years of age	3,879	Mobile phone-based application providing information about oral health care for children from infancy to 6 years of age	Significant improvement in mothers’ knowledge; especially those with more than one child.
Zolfaghari, M., et al. (2021) [79]	Iran	Mothers of preschool children	58 mother and child pairs	Mobile phone application containing information about ECC, healthy diet, sugars, baby-oral hygiene, fluoride effect, fluoride toothpaste, and toothbrushing training video	Improved oral-health knowledge and practice of mothers.
Lai, B., et al. (2018) [80]	Singapore	Children under 18 months of age and their caregivers	90	Oral health education, anticipatory guidance on diet, oral health care practices, non-nutritional habits and trauma prevention, and topical fluoride varnish	Reduction in SECC among infants and toddlers.
Jamieson, L., et al. (2018) [81]	Australia	Pregnant women and their children	448	Dental care to mothers during pregnancy, application of fluoride varnish to teeth of children at ages 6, 12, and 18 months. Motivational interviewing delivered in conjunction with anticipatory guidance	Improvements in the oral health of children.
Wagner, Y., et al. (2014) [82]	Australia	Mothers at time after birth	471	Dental health educators visited all mothers at a time after birth and provided comprehensive oral hygiene instructions for their children and themselves, including practical toothbrush training and dietary counseling by the use of brief motivational interviewing and anticipatory guidance approaches	Children showed significantly lower caries prevalence and experience.
Neumann, AS., et al. (2011) [83]	Australia	Families with children aged 7–8 months	915	Community-based intervention by local maternal and child health nurses. The intervention includes the provision of an age-appropriate toothbrush, toothpaste, and educational information for parents	Reduction in caries prevalence in the second year of life but less so in older children.
Ismail, A., et al. (2018) [84]	Malaysia	2–3 y children, 4–6 y siblings, and their mothers	478 mother-child-sibling trios	Oral health education through anticipatory guidance at six-month intervals over 3 years.	Reduction in children’s and siblings’ caries incidence and improved mother’s oral health literacy.
Henshaw, M., et al. (2018) [85]	USA	0–5 y children and their caregivers	1,065	Motivational interviewing with a focus on ECC prevention, quarterly clinical examinations, fluoride varnish applications, toothbrush, toothpaste, and educational brochures	Counselling and intensive caries prevention activities resulted in knowledge increases but did not improve oral health behaviors or caries increment.
Macintosh, AC., et al. (2010) [86]	Canada	Service providers and community members who work with infants, preschool children, and their families	108	community workshops targeting participants with early childhood oral health knowledge and ECC prevention	Capacity-building workshops increased oral health knowledge and self-reported behaviours.
Anderson, M., et al. (2016) [87]	Sweden	1 y children	3,403	Semi-annual professional applications of fluoride varnish until the age of three	The intervention had no additive effect in reducing the prevalence of ECC.
Tickle, M., et al. (2017) [88]	Ireland	2–3 y children	1,248	Fluoride varnish, toothbrush, fluoride toothpaste, evidence-based prevention advice	The intervention failed to Keep children caries free, however, there was evidence that once children get caries it slowed down its progression.
Memarpour, M., et al. (2016) [89]	Iran	1–2 y children and their mothers	300	Oral health counseling and fluoride varnish	Oral health counseling alone or associated with the use of fluoride varnish reduced the caries incidence
Macpherson, LM., et al. (2019) [90]	Scotland	3–4 y children	A Scotland-wide population	Supervised toothbrushing at nurseries. Children are provided with a dental pack containing a toothbrush and toothpaste. Children also receive fluoride varnish	The program succeeded to increase the number of children with no caries experience and reduce the dmft index.

## Discussion

This scoping review provided an overview of the global trends of ECC prevalence with more focus on the GCC region. It also highlighted many ECC preventative programs around the globe and identified the need for further actions to efficiently utilize available resources within the GCC region to tackle the disease. This review included studies utilized both the WHO dmft index as well as International Caries Detection and Assessment System (ICDAS) for dental caries assessment. For cavitated carious lesions, both indices tend to report similar prevalence rates. Nevertheless, dmft index is unable to detect non-cavitated lesions cases, unlike the ICDAS which has the advantage of distinguishing between the stages of caries progression in early enamel, enamel, and dentin [91, 92]. Hence the variation between the two indices needs to be taken into consideration while interpreting the results of this review.

### Global trends of ECC prevalence

Dental caries is the most prevalent chronic disease in early childhood [5]. The prevalence of ECC varies globally according to the population studied. Socially disadvantaged groups in communities around the world are affected most by the disease [93, 94]. The prevalence of dental caries has shown a marked decrease over the last quarter of the twentieth century in industrialized countries. This is due to various public health measures such as water fluoridation and other fluoride modalities along with changing living conditions and improved self-care practices [95]. In contrast, dental caries is still considered a major health problem worldwide [96]. Certain disadvantaged groups in industrialized countries and many other populations in developing countries are suffering from an increasing prevalence of dental caries [96].

### Risk factors of ECC with a focus on the GCC region

According to the WHO *“The social determinants of health are the conditions in which people are born*, *grow*, *live*, *work and age*. *These circumstances are shaped by the distribution of money*, *power*, *and resources at global*, *national*, *and local levels*. *The social determinants of health are mostly responsible for health inequities—the unfair and avoidable differences in health status seen within and between countries”* [97].

It is generally agreed there are many individual risk factors and social determinants for dental caries. The individual risk factors include early acquisition of caries-causing bacteria, *mutans streptococci*, and consuming a highly cariogenic (causing caries) diet. Poor dietary habits play a significant role in caries development including bottle feeding beyond 15 months of age, bottle feeding in bed, prolonged on-demand and frequent breastfeeding, and continuous sipping from the bottle during the day [98]. Other risk factors include lack of fluoride exposure in the absence of water fluoridation, less than daily tooth brushing, tooth enamel abnormalities, and limited professional guidance from medical practitioners, dentists, pediatricians, and nurses [99–102].

There might be some risk factors for ECC that are unique to the GCC region in comparison with many places in the world. In general, the population in the GCC region is characterized by relatively high income per capita by international standards. However, ECC remains a significant problem in this region coupled with a lack of water fluoridation in most GCC countries. In the following sections, reported risk factors which are relevant to the GCC region will be discussed.

#### Parental knowledge and attitudes

Upon reflection on the unique situation of the GCC countries, interesting findings were observed. A study that investigated the knowledge of mothers of preschool children about oral health in Qatar reported good knowledge about oral health care. However, the study reported that 36% of the children went to bed with a bottle and 42% of the children have frequent snacks which are mostly cariogenic. These findings indicate that mothers were unable to translate their knowledge into a habit [103]. Similar findings were reported by a study from Kuwait investigating the knowledge, attitudes, and practices of caregivers in relation to the oral health of preschool children [13]. The participants in this study had good knowledge about inappropriate types of diet that may cause dental caries, the importance of fluoride, and its role in preventing dental caries. However, their knowledge and attitude scores were low about the timing of starting toothbrushing for young children, the timing of the first dental visit, and the consequences of ECC. More than two-thirds of the participants could not indicate the correct age for the first dental visit and reported their children to have visited the dentist after the age of 3 years. The most interesting practice reported in this study was that around 44% of the children have their teeth brushed by a caregiver other than the parents. It is worth mentioning that having nannies to take care of the children is a common practice in the GCC region. Two other studies from the UAE reported that the level of parents’ education had a significant association with the occurrence of dental caries in their children [104, 105].

#### Nationality

Interestingly, in the GCC region, being national or indigenous can be a risk indicator for dental caries. Hashim *et al*. [104] reported a caries prevalence of 76% and an average dmfs score of 10.2 in a study conducted in UAE, involving 1036 children aged 5–6 years. In this study, the authors found that native children had more severe caries than expatriates.

### ECC management and prevention

There are many strategies to manage and prevent ECC that have been identified in the literature. These strategies include the early establishment of a dental home, periodic examination and preventive practices, anticipatory guidance, and treatment when necessary. It is recommended that children should have their first dental examination no later than 12 months of age by a child-friendly dental practitioner. This is referred to as establishing a “dental home” [106–108]. A periodic dental examination is recommended at 6-month intervals and maybe more frequent if the child was identified as having a high dental caries risk. During this periodic examination, dental professionals may undertake preventive practices including caries risk assessment, prophylaxis, and topical fluoride treatment as well as providing fluoride supplementation when needed [109, 110]. In early childhood, anticipatory guidance is appropriate in areas such as oral hygiene, dietary habits, injury prevention, and speech and language development. Early detection of oral disease and providing the appropriate and optimal treatment complete the strategic approach to ECC management [111, 112].

### Overview of ECC oral health prevention programs

There are a variety of oral health promotion programs that have been researched for the impact and effectiveness of preventing ECC. Those intervention programs suggested oral health education, nutritional programs, the use of fluorides, sugar substitutes, and mechanical barriers such as pit-and-fissure sealants. Behavioral interventions such as motivational interviewing, anticipatory guidance, and counseling with children and their caretakers are also suggested [113]. The following sections shall discuss some of the oral health prevention programs implemented in different counties to prevent ECC.

#### GCC countries

A school-based oral health promotion program was implemented in Kuwait, targeting 11–12-year-old children. The program was evaluated by Alsumait A, et al. [77] who included 440 primary school children who participated in the program for at least 3 years and compared their oral health status with a control group. During the program, children received twice-a-year applications of fluoride varnish and fissure sealants if needed; and their mothers had, at least, one oral health education session. The results indicated that enrolment in the school-based prevention services was associated with a positive impact on children’s caries level with no significant impact on mothers’ knowledge.

In Qatar, a school-based oral health program was established in 1979 and was redesigned in 2017 to a new program targeting kindergarten and all grades of primary public schools. The program provides age-specific oral hygiene instructions where children are engaged in educational and recreational activities, application of topical fluoride varnish, and referral to a primary healthcare center as needed [114].

Technologies have been utilized to implement some oral health promotion programs such as mobile phone applications sought to enhance oral health knowledge and practice of mothers. In the Kingdom of Saudi Arabia, a mobile application has been developed and distributed to 3879 mothers of children below 6 years of age. The impact of the intervention was studied and the authors reported that there was a significant improvement in the knowledge of the mothers; especially those with more than one child [78]. Similar findings were reported from Iran by Zolfaghari M, et al., [79], where a mobile phone application was developed containing information about ECC, healthy diet, sugars, baby-oral hygiene, fluoride effect, fluoride toothpaste, and toothbrushing training video.

#### Hongkong

Chai HH, et al., [115] implemented a school-based oral health promotion program where they targeted 20,000 children from 100 kindergartens in Hong Kong with dental screening and silver diamine fluoride treatment to manage ECC. In addition, oral health talks were given to the children’s parents, and teacher training was provided to empower teachers to deliver regular oral health education to kindergarten children at school. The project also provided individual counseling to parents whose children have severe ECC.

#### Singapore

A two-year quasi-experimental study included 90 children under 18 months of age and their caregivers in Singapore. The study evaluated a preventive oral health program including oral health education, anticipatory guidance on diet, oral health care practices, non-nutritional habits and trauma prevention, and topical fluoride varnish. The results indicated that 31.3% of children in the control group had SECC compared to 7.8% in the intervention group [80].

#### Australia

A randomized controlled trial from Australia evaluated an oral health promotion program that offered dental care to mothers during pregnancy, application of fluoride varnish to teeth of children at ages 6, 12, and 18 months and motivational interviewing delivered in conjunction with anticipatory guidance. The mean number of decayed teeth in children aged two years was lower in the intervention group (0.62) than in the control group (0.89) [81]. Another community-based oral health prevention in Australia improved oral health in preschool children where dental health educators visited all mothers at a time after birth and provided comprehensive oral hygiene instructions for their children and themselves, which included practical toothbrush training and dietary counseling by the use of brief motivational interviewing and anticipatory guidance approaches [82].

The “Country KIDS” study was conducted in rural Victoria, Australia, and investigated the effect of a community-based intervention promoting early exposure to fluoridated dentifrice [83]. This study involved healthy children (n = 915) from three rural areas who were recruited into the study by the maternal and child health nurses at the age of 12 months. The nurses received oral health promotion training as part of the study. The intervention group received an oral health starter kit containing toothpaste, toothbrush, and educational materials about oral health, while the control group received standard care. Children were examined at baseline and then annually for three years. The results of this study were inconclusive.

#### Malaysia & The USA

In Malaysia, the Family Dental Wellness Programme is implemented targeting 2–3-year-old preschool children and their 4–6-year-old siblings’. The program offered dental examinations and oral health education through anticipatory guidance at six-month intervals over 3 years and it significantly lowered net caries increment among enrolled children [84]. On the other hand, a community-based cluster-randomized controlled trial conducted in Boston—The United States of America (USA) concluded that motivational interviewing counseling with intensive caries prevention activities resulted in knowledge increases but did not improve oral health behaviors or caries increment among children aged 0 to 5 years [85].

#### Canada

Another study evaluated the impact of community workshops on improving knowledge about ECC in Manitoba, Canada. The participants (n = 108) in this study were service providers and community members who work with infants, preschool children, and their families. They were engaged in capacity-building workshops and participated in a pre-and post-workshop survey to assess the effectiveness of the workshops. Many participants had good prior knowledge that foods high in sugar and starch cause dental caries. However, they had limited prior knowledge about the initial signs of dental caries, the timing of the first dental visit for children, the extent of parents’ supervision for child toothbrushing, and the adverse effect of mother’s poor oral health on her child’s oral health and the protective role of fluoride varnish in preventing dental caries. These areas of poor knowledge were reported to be improved by 16% after the workshops [86].

#### Sweden & Ireland

A study from Sweden evaluated semi-annual professional applications of fluoride varnish in one-year-old children until the age of three. The program failed to reduce caries development [87]. Similar findings were reported by Tickle M, et al., [88] who evaluated the effect of topical fluoride application in 2-3-year-old children over three years in Ireland. In contrast, a one-year clinical trial conducted in Iran targeted children aged 12–24 months with oral health counseling and fluoride varnish and concluded that oral health counseling alone or associated with the use of fluoride varnish reduced caries incidence [89].

#### Scotland

Scotland implemented a national comprehensive oral health prevention program “Childsmile”. The program sought to improve the oral health of children as well as access to dental services. The program offers supervised toothbrushing at nurseries. Moreover, children are provided with a dental pack containing a toothbrush and toothpaste. Children also receive fluoride varnish by Childsmile dental nurses. The program succeeded to increase the number of children with no caries experience in primary teeth from 45% in 2003 to 71% in 2018. In addition, the dmft index is reduced from 2.76 to 1.14 during the same period of time [90].

### The role of medical practitioners in children’s oral health

Evidence from the scientific literature on ECC prevention highlights the need for early engagement of parents perinatally. The involvement of non-dental healthcare providers in ECC prevention programs is considered efficient and cost-effective. Hence, inter-professional population-based oral health promotion and prevention programs hold the potential to target children at greatest risk and address their oral health within a larger context of overall health [113].

A publication by the Victorian Department of Health in Australia provided a comprehensive review of health promotion activities [116]. This review presented oral health promotion interventions for different age groups including pregnant women, babies, and young children. The authors suggested that successful oral health promotion programs share common elements: integrating oral health into general health programs, the use of fluoride, targeting high-risk populations, tailored approaches based on active participation and addressing social, cultural, and personal norms and values and the existence of surveillance and referral targeting pregnant women, infants, and young children. Hence the integration of oral health into well-child visits was through The *Lift the Lip* screening program. The program was adopted by primary healthcare centers in Qatar in 2014, where around 1000 non-dental health professionals (nurses, physicians, and health educators) were trained in oral health promotion and simple oral examination to detect caries and other oral health problems. The program is called “The Beautiful Smile Project” where young children visiting the vaccination clinics (Well-Baby Clinics) are screened by attending nurses and physicians for oral diseases including dental caries and referred to a dental clinic as needed. Moreover, nurses and physicians attending antenatal clinics provide oral health checks for expectant mothers and refer them to the dental clinic as needed [117].

## Conclusion

This review mapped the prevalence of ECC in many countries across the world and gave an insight into the disease prevalence in the GCC region in relation to other geographical areas. ECC remains a significant problem in the GCC region despite the implementation of several oral health prevention programs highlighted in this review. It is evident that the GCC region has unique characteristics that need further investigations to effectively contextualize the western model of the dynamics of ECC prevention and promotion programs locally. Hence further research is needed in this area to inform policymakers on how to effectively utilize resources to tackle the disease.

## Supporting information

S1 Checklist Preferred Reporting Items for Systematic reviews and Meta-Analyses extension for Scoping Reviews (PRISMA-ScR) checklist. (DOCX)
